# Supplementary figures and images for: Single- and duplex TaqMan-quantitative PCR for determining the copy numbers of integrated selection markers during site-specific mutagenesis in Toxoplasma gondii by CRISPR-Cas9
Source: PLoS One. 2022 Sep 16;17(9):e0271011. doi: 10.1371/journal.pone.0271011 (PMC9481009; doi:10.1371/journal.pone.0271011)

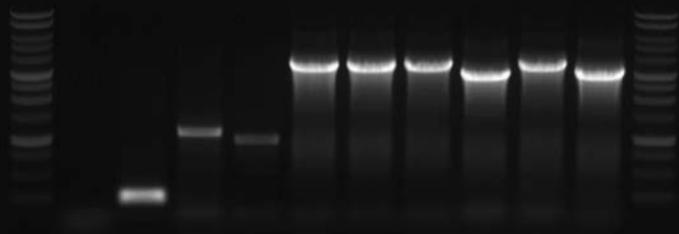

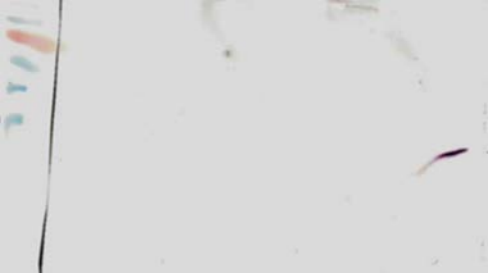

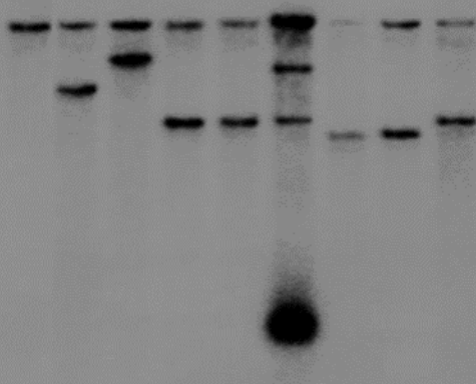

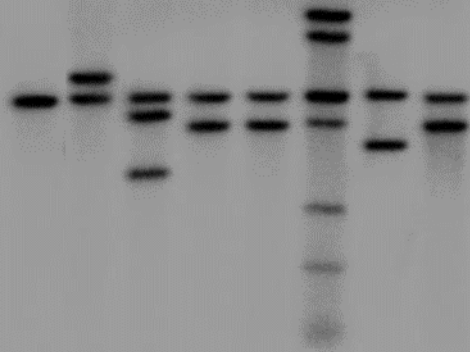

Supplement: S1 Raw images — (PDF) [file pone.0271011.s001.pdf]
