# Supplementary material for: Single- and duplex TaqMan-quantitative PCR for determining the copy numbers of integrated selection markers during site-specific mutagenesis in Toxoplasma gondii by CRISPR-Cas9
Source: PLoS One. 2022 Sep 16;17(9):e0271011. doi: 10.1371/journal.pone.0271011 (PMC9481009; doi:10.1371/journal.pone.0271011)
Supplement: Attachment — Submitted filename: response letter.docx [file pone.0271011.s003.docx]

**Response letter**: **PONE-D-22-17694** “Single- and duplex TaqMan-quantitative PCR for determining the copy numbers of integrated selection markers during site-specific mutagenesis in *Toxoplasma gondii* by CRISPR-Cas9”.

------------------

**Dear Editor,**

Sincerest thanks for your response and reviewers for their time and their constructive suggestions and comments. We have revised the manuscript accordingly and we hope that these changes comply with the referee’s critics and suggestions.

**Reviewer 1**

The authors described a methodology to follow the mutations generated by CRIS Cas 9, for this they invalidated the *Toxoplasma* SAG1 gene and then used qPCR duplex to demonstrate the insertion of mutations. In general, the authors describe appropriately their methodology and results. The results are convincing, and they discuss the reach and limits of the method. I have only some minor suggestions to improve clarity for readers:

**Rev1-1.** They put in discussion something that should be described in results: the rate of success of mutated clones achieved in 5 of 33 clones (15%).

**→ Response:** we agree and we have first cited this information in the result section (line 273 – 274) and then discussed it (line 404 – 407).

**Rev1-2.** One of the most critical and important results is the demonstration that SAG1 expression was invalidated as it was showed by western blot and IFAT experiments, this should NOT be supplementary material, please include as Figure 1.

**→ Response:** we have now moved the supplementary Figure 1 showing the IFA and WB to the main result section; subsequently it is now cited as Figure 2 (in the revised version). We have accordingly changed the numbering of figures: for example, figure 2 is now figure 3 in the revised version etc.

The IFA and WB was not included as Figure 1, because we followed the order of the experimental steps; we first did the diagnostic PCR and subsequently we checked for SAG1 expression.

**Rev1-3.** The formula showed in Figure 4 please show as text in the section of results

**→ Response:** In the revised version, Figure 4 is Figure 5; we included in the text (line 332 – 335) the formula expressing principle and potential outcome of the duplex TaqMan-qPCR.

**Reviewer 2**

The work by Haenggeli et al aims to describe single or duplex qPCR for the quantification of a mutated version of DHFR integrated as a drug marker during CRISPR/Cas9 genome editing in *Toxoplasma gondii*.

The manuscript is well written and scientifically sound. The authors developed a technique that estimates in an accurate manner if a commonly used drug marker for positive selection during genetic depletions in the *Toxoplasma* model is correctly inserted in the intended genomic locus, as well as if there were any off target insertions of multiple drug markers.

**Rev2-1.** The insertion of multiple drug markers is a rare event nowadays, especially due to a highly efficient and targeted CRISPR/Cas9 double stranded break - as shown by the authors, which yielded a single clone with that caveat out of 33 analyzed. Also, although multiple off target insertions are not desired, it usually does not have a phenotypic impact on the parasites, unless multiple other genomic editions are planned - and, still, it is unlikely to alter the planned outcome. Nonetheless, the protocols described are truly useful for researchers of the area that may be concerned with such ‘side effects’ of genomic editing.

**→ Response:**

Although reviewer 2 acknowledged the usefulness of this technique, we do not entirely agree with the statement that multiple insertions, when occurred, usually they do not have a phenotypic impact of the desired Knockout. This is for the following reasons:

- First, there is no study in *Toxoplasma* showing that multiple insertion events of a selectable marker have no effect on the phenotype of a planned knockout. Currently CRISPR-Cas9 is applied in most of functional genomics published studies and the use of *Toxoplasma* ∆Ku80 strains is still dominant. This is to ensure repair of double strand DNA breaks via homologuous recombination by introducing a selection gene flanked by homology arms to the 5' and 3' DNA sequences of the targeted gene. This strategy aims at avoiding multiple insertion events. Thus, if it would be true that multiple insertions are of no impact, we would not try to avoid them using *Toxoplasma* ∆Ku80.
- Second, for example in the case of the DHFR-TS selection marker, this gene, once integrated in the genome, is expressed as a fully active enzyme DHFR-TS which participates in the folate and thymidylate biosynthesis, both required for DNA synthesis and repair. Thus in case of multiple insertions, particularly when occurring in coding genes, the levels of DHFR-TS are higher than in case of single integration, which is likely to impact on folate pathway activity. The same accounts for other selection markers.
- Third, multiple insertions can impact on genetic complementation toe be used for validation of the knock-out phenotype. This may explain partially the failure of a complemented knockout to recover the wild-type phenotype.

**Rev2-1. Minor comments**

The authors write several lines on how Ku80 strains have positively impacted the field, allowing and increment in transfection efficiency in *Toxoplasma*. However, they use wild type RH strain tachyzoites in their study. They even argument that, with their technique, WT parasites could be used more frequently in genomic editing protocols instead of base strains with genomic depletion of the ku80 gene. Why?

**→ Response:**

- Indeed *T. gondii ∆ku80* strains exhibit several technical advantages for genetic manipulation of *T. gondii*, nonetheless from the biological point of view they are genetically modified parasites.

- As outlined in the introduction and discussion, *Δku80* parasites are deficient in the non-homologous end-joining pathway required for repairing DNA double-strand breaks. Genetic manipulation in *T. gondii* occurs using the tachyzoites proliferative stage. During continuous *in vitro* culture of tachyzoites, and many cycles of DNA replication/division, it is conceivable that mutations, strand breaks and other events may occur in the genome. In case of *∆ku80* strains, these modifications are accumulating, irreversible and irreparable due to the lack of the Ku 80 enzyme, a main component of the NHEJ. Thus, an observed phenotype/ effect following the deletion/interruption of a gene X can be impacted by the first mutation (*∆Ku80*) as explained in the figure above (**a**).
- In addition, as shown in the Figure (**b**), when using *∆ku80* strains for gene knockout or knockin, it is not correct to extrapolate the results to the circulating wild type genotypes, which do not have any defect in NHEJ. Deducing the impact of the introduced mutation on wild type *Toxoplasma* strains is hampered by the impracticality of subtracting the effect of the absence of the Ku80; at a time, this effect also cannot be ignored.

**Rev2-2. Minor comments**

Are WT parasites known to generate multiple off target incorporations of the drug markers? To my knowledge, the advantage of transfecting ku80 KO parasites is the increased efficiency of the incorporation of foreign DNA, not avoiding multiple insertions of drug markers. Please indicate/discuss with proper references on those affirmatives or remove them from the text.

**→ Response:**

The advantage in using *∆ku80* parasites was to ensure that homologous recombination is the only mechanism used to repair the double-stranded DNA breaks. This strategy allows to completely avoiding the NHEJ pathway, which is the dominant repair pathway in WT *Toxoplasma* and causes multiple random integration of the exogenous DNA*.*

Thus, before the establishment of CRISPR-Cas9, the rational in using *∆ku80* instead of WT parasites is to increase the efficiency of occurrence of the targeted mutation (insertion of the selection cassette in the targeted gene). In another way, it is more about targeted integration (∆ku80 parasites) versus multiple/random integration (WT) **BUT** it does not concern the efficiency of exogenous DNA incorporation.

***►In the manuscript****, particularly in* ***the introduction*** *[****Line 62-71****] we stated the rationale behind using ∆ku80 parasites; which is to* ***increase the efficiency of occurrence of the targeted mutation****.*

CRISPR-Cas9 has improved specificity because the gRNA derives from the targeted gene, subsequently WT *Toxoplasma* parasites can be also used. In this case, the NHEJ pathway is engaged/oriented to repair the specific DNA break, which occurred in the gene of interest.

***►In the manuscript****, particularly in the introduction [****Line 79-86****] we explained how* **CRISPR-Cas9** allowed the use of WT parasite.

**Rev2-3. Minor comments**

- Minor revision of the text is desirable to eliminate certain formatting errors (e.g. references on line 58; lack of an additional espace on line 231; parenthesis on line 329; and so on…)

**→ Response:**

This was an error: references on line 58 was corrected there are now toxoplasmosis [5,6]. A space was included in line 231. We changed the parenthesis in line 329 (in the revised line 340) from superscript to regular script.

**Rev2-4. Minor comments**

- Lines 282-288: Paragraph should be revised for clarity: Sanger sequencing is not demonstrated; insertion of “short DNA sequence” or integration of mDHFR-ts elsewhere also is not proven properly.

**→ Response:**

In the revised version (line 288), we briefly indicated that in clone C6 and 7, the inserted DNA in the *sag1* locus were sequences from the Plasmid 926 (Cas9/gRNA plasmid); probably some degraded sequences of the plasmid and also from *mdhfr-ts* were used as template to repair the DNA break in *sag1*.

We show here the sequence of C6, however we did not include this in the manuscript since we only used this clone as control knockout having a single integrated *mdhfr-ts* not in the *sag1* locus but elsewhere in the genome. This to demonstrate that single and duplex TaqMan-qPCR performs a full scan to detect *mdhfr-ts* independently of its location.
